# Successful Vaginal Delivery in a Pregnant Woman With Bernard–Soulier Syndrome: A Case Report

**DOI:** 10.1002/ccr3.71706

**Published:** 2026-02-08

**Authors:** Monireh Yaghoubi, Nona Sabeti, Ezat Hajmollarezaei

**Affiliations:** ^1^ Department of Obstetrics and Gynecology, Faculty of Medicine Mashhad University of Medical Science Mashhad Iran; ^2^ Supporting the Family and the Youth of the Population Research Core, Department of Obstetrics and Gynecology, Faculty of Medicine Mashhad University of Medical Science Mashhad Iran

**Keywords:** Bernard–Soulier Syndrome, bleeding disorder, case report, platelet dysfunction, pregnancy

## Abstract

Bernard–Soulier syndrome (BSS) is a rare inherited platelet disorder that poses major risks during pregnancy because of potential bleeding complications. Managing such cases in low‐resource settings is especially challenging. We report the case of a 23‐year‐old Afghan woman with BSS who achieved a successful full‐term vaginal delivery at a tertiary center. Early referral, coordinated management by hematology and obstetric teams, prophylactic platelet transfusions, and prompt treatment of postpartum hemorrhage led to a favorable maternal and neonatal outcome. This experience shows that, even where advanced laboratory and transfusion resources are limited, structured planning, timely communication, and preparedness can ensure a safe delivery for women with complex hematologic disorders.

## Key Clinical Message

A multidisciplinary strategy combining prophylactic platelet transfusion, tranexamic acid, and uterotonic agents enabled a safe vaginal delivery in a patient with Bernard–Soulier syndrome, indicating that cesarean section is not the only option in such high‐risk hematologic conditions.

## 1 Introduction

Bernard–Soulier Syndrome (BSS) is a rare inherited platelet disorder that impairs the clotting ability of platelets [1]. This condition arises from either a quantitative or qualitative deficiency in the glycoprotein (GP) receptor complex embedded in the platelet membrane. BSS is heterogeneous and classified into three types based on the residual expression of the GPIb‐IX‐V complex. Type 1 (< 5% expression) represents the most severe phenotype with a high‐risk of bleeding [2, 3]. As the primary receptor for von Willebrand factor (vWF), this complex plays a crucial role in platelet adhesion and aggregation. The disorder is inherited as an autosomal recessive trait, leading to significant abnormalities in platelet function and hemostasis [4]. BSS affects both sexes equally, as it is inherited in an autosomal recessive manner. Common symptoms include easy bruising, gum bleeding, heavy menstrual bleeding, recurrent nosebleeds, gastrointestinal bleeding, and excessive bleeding following trauma. The severity and presentation can vary, often necessitating careful management to prevent complications [5].

Pregnancy can amplify the risk of bleeding in individuals with BSS due to an increased demand on the coagulation system. Reported rates of PPH vary and are often based on patients with severe phenotypes; data for milder cases remain limited [6]. Furthermore, severe thrombocytopenia was noted in approximately half of these pregnancies, and antepartum hemorrhage was observed in several cases [7].

Despite these potential complications, literature on the management of pregnancy and childbirth in women with BSS remains sparse. This case report describes the successful delivery of a healthy baby by a 23‐year‐old Afghan woman diagnosed with BSS. This case report has been reported in line with the SCARE checklist [8].

## 2 Case History/Examination

A 23‐year‐old Afghan woman at 38 weeks and 2 days of gestation was referred to Ghaem Hospital, affiliated with Mashhad University of Medical Sciences, for pregnancy management. She was a primigravida (G1P0) with no history of miscarriage or stillbirth. She had a known history of BSS, diagnosed at 3 months of age. Formal bleeding assessment scoring was not performed due to limited access to the ISTH‐BAT, but clinical history suggested a severe phenotype. Based on her history, she would likely score in the severe range. Her bleeding tendency was managed using intermittent tranexamic acid and occasional platelet transfusions for significant epistaxis or surgical bleeding. Despite relatively stable disease management, she continued to experience two to three episodes of menorrhagia annually. At the age of 17, she underwent a successful laparotomy for the removal of a hemorrhagic ovarian cyst, followed by a cystectomy.

During her pregnancy, under the joint care of a hematologist and gynecologist, the patient required four platelet transfusions due to a persistently low platelet count. Despite her condition, she had no hemorrhagic events throughout the pregnancy. Upon admission to the hospital, she presented with no vaginal bleeding, uterine contractions, or leakage of amniotic fluid. Fetal movement was normal, and her vital signs and physical examination findings were within normal limits. She received leukoreduced, irradiated, single‐donor apheresis platelets that were cross‐matched before transfusion. The four platelet transfusions were prophylactic, administered due to persistently low platelet counts in the context of Bernard–Soulier Syndrome, aiming to minimize the risk of spontaneous bleeding and to ensure maternal and fetal safety. No transfusion reactions were observed during or after these transfusions.

Laboratory investigations showed a hemoglobin level of 13.9 g/dL, a hematocrit of 40.2%, and a platelet count of 50,000/μL. Coagulation parameters, including prothrombin time (PT), partial thromboplastin time (PTT), and international normalized ratio (INR), were within normal ranges.

Labor was induced by the insertion of a cervical catheter and the administration of 25 micrograms of misoprostol sublingually. Tranexamic acid (1 g IV) was administered prophylactically at induction of labor, followed by intermittent dosing post‐delivery to minimize bleeding risk. No prophylactic platelet transfusion was administered at induction, as the platelet count was 50,000/μL and the patient had no bleeding. Platelet transfusion was initiated immediately after delivery when postpartum hemorrhage developed. The patient progressed with spontaneous contractions and delivered a healthy baby girl, weighing 2265 g, after less than 2 h of active labor. The newborn had Apgar scores of 9 and 8 at 1 and 5 min, respectively. Immediately after delivery, the patient received 20 units of oxytocin in 1000 mL of Ringer's solution, followed by 600 micrograms of misoprostol sublingually as prophylaxis for postpartum hemorrhage (PPH).

## 3 Methods

Despite the administration of pharmacological interventions, the patient developed hemorrhage and was swiftly transferred to the operating room for further examination and management under anesthesia. The hemorrhage occurred within 1 h after delivery, thus classified as early postpartum hemorrhage. The patient experienced transient hemodynamic instability (tachycardia up to 120 bpm and mild hypotension) but did not progress to full hypovolemic shock due to prompt fluid and blood resuscitation. During the exploratory procedure, a revision of the uterus was performed and a grade 2 episiotomy and a 3 cm laceration on the right lateral wall of the vagina were repaired. In response to uterine atony, a Foley catheter was placed. The patient received 5 units of factor 7, 10 units of platelets, 2 units of apheresis platelets, 2 units of fresh frozen plasma (FFP), and 3 units of packed red blood cells (PRBCs) during the operation. Following the procedure, she was moved to the Intensive Care Unit (ICU). The transfusion strategy in the ICU prioritized platelet replacement due to the qualitative platelet dysfunction inherent in Bernard–Soulier Syndrome. The relatively high proportion of platelets administered reflected both refractoriness to random donor platelets and the need to maintain adequate hemostasis during surgical recovery. When available, single‐donor apheresis platelets were preferred to minimize alloimmunization risk.

During her ICU stay, the hematology team managed the patient's coagulation status using 10 units of platelets, 2 units of FFP, 5 units of recombinant factor seven, and 3 units of packed cells. After her coagulation status was stabilized, she was discharged from the ICU after 4 days. Given the hereditary nature of BSS, we screened the neonate. Her platelet count was normal (150,000/μL), which ruled out BSS.

## 4 Conclusions and Results

Our case indicated that despite the high‐risk of hemorrhagic complications associated with BSS, favorable maternal and neonatal outcomes can be managed with a planned, multidisciplinary approach. The patient underwent vaginal delivery and postpartum hemorrhage was effectively managed with prompt surgical and hematologic interventions. She was discharged in a stable condition after intensive care in the ICU. The absence of BSS was confirmed by postnatal screening, highlighting the importance of neonatal evaluation due to the hereditary nature of this condition.

In conclusion, managing BSS during pregnancy presents unique challenges due to the increased risk of bleeding. However, with meticulous planning and a multidisciplinary approach, successful pregnancy outcomes can be achieved. The importance of prophylactic measures against bleeding, carefully planned delivery modes, and rigorous postpartum monitoring cannot be overstated. Although a favorable outcome was achieved, the case underscores that significant bleeding can still occur despite planned management, highlighting the need for stronger prophylactic measures and early referral.

## 5 Discussion

A comprehensive literature review reported a 52.9% incidence of postpartum hemorrhage (PPH) in deliveries involving BSS, with late PPH occurring more frequently than early PPH. The study also indicated a lower risk of PPH in women who received peripartum prophylaxis, suggesting that preventive measures may play a crucial role in reducing bleeding complications in BSS pregnancies [6]. Our case aligns with these findings, as our patient who had a history of recurrent epistaxis, menorrhagia, and a hemorrhagic ovarian cyst developed the expected postpartum hemorrhage. However, timely prophylactic measures and rapid intervention ensured effective management, preventing further complications such as urgent hysterectomy.

Despite the scarcity of BSS, Sarıdoğan et al. indicated that 64.7% of BSS patients underwent cesarean sections, with PPH more common in those who had vaginal deliveries [6]. Conversely, our patient achieved a successful vaginal delivery after induction with a cervical catheter and misoprostol. Since there is insufficient evidence to establish the superiority of any specific mode of delivery in BSS patients, management should be tailored to each case individually [9]. However, with proper preparation and careful monitoring, vaginal delivery can be safely achieved, emphasizing the importance of a patient‐centered approach in optimizing maternal and neonatal outcomes. While prophylactic platelet transfusions were administered during pregnancy based on persistently low platelet counts, the timing and number of transfusions may not have been sufficient to completely prevent postpartum hemorrhage. The decision for vaginal delivery was made to minimize surgical trauma and avoid potential complications of cesarean section and anesthesia. However, this case underlines that even with careful planning, PPH remains a significant risk and requires immediate preparedness for surgical and hematologic intervention.

In managing BSS during pregnancy, epidural anesthesia is generally advised against due to heightened bleeding risks, and this should be communicated during prenatal visits [10]. In this patient, neuraxial anesthesia was avoided and pain control was achieved with acetaminophen and opioids; aspirin and NSAIDs were strictly avoided. A trial of labor was carefully planned to minimize the risks associated with platelet depletion and excessive bleeding that can occur during a Cesarean section. Additionally, opting for vaginal delivery aimed to reduce the likelihood of hematoma formation and hemorrhage at the site of regional anesthesia. Scalp monitoring during labor is not recommended, and the use of forceps is discouraged. In this case, all invasive fetal monitoring (e.g., scalp electrode) and operative vaginal delivery techniques (forceps or vacuum) were strictly avoided to reduce neonatal bleeding risk. A thorough neonatal assessment is essential, including monitoring for signs of bruising or bleeding, and if the syndrome is suspected, a cranial ultrasound is necessary to check for intracranial hemorrhage. Instead of the heel prick test and intramuscular vitamin K, oral vitamin K is preferred. While platelet transfusions have been used for both active bleeding treatment and prophylaxis before delivery, the benefit of prophylactic transfusions remains unclear, highlighting the need for further research in this area [10]. Our approach, using leukoreduced, single‐donor, cross‐matched platelets in a prophylactic manner, is in line with recommendations by Huisman et al., who propose selective HLA‐ or antigen‐matched platelet transfusion strategies in patients with congenital platelet disorders to mitigate alloimmunization risk. However, in our setting, full HLA‐matching was not feasible due to resource constraints, so we prioritized leukoreduced and cross‐matched products as a pragmatic compromise. We also acknowledge that we could not perform corrected count increment (CCI) assessments due to laboratory limitations, relying on clinical outcomes instead [11]. Exploring alternative therapies, such as antifibrinolytic therapy (tranexamic acid) and activated factor VII, may also yield promising results [12].

This case is unique because it was managed in a resource‐limited setting, where access to HLA‐matched platelets, recombinant factor VIIa, and specialized hematology support is often challenging. Despite these limitations, the multidisciplinary team successfully planned a vaginal delivery, promptly controlled postpartum hemorrhage, and avoided hysterectomy. These aspects distinguish this report from most published cases, which commonly describe cesarean deliveries or management in highly equipped centers. Our findings demonstrate that favorable outcomes can be achieved even in low‐resource environments when timely referral and coordinated care are ensured. Besides, our case reinforces the importance of a multidisciplinary team approach in managing BSS during pregnancy. Collaboration between hematologists, obstetricians, and anesthesiologists is crucial to anticipate and manage complications and optimize outcomes for both the mother and neonate. Furthermore, this case highlights the significance of neonatal BSS screening due to its hereditary nature.

Given the diverse clinical presentations of BSS, each case must be evaluated individually and managed according to the specific needs and condition of the patient. Tailored treatment strategies, based on patient history, severity of symptoms, and personal preferences, are essential. The potential risks associated with each mode of delivery must be thoroughly evaluated and communicated to the patient.

In the face of such complexity, the importance of ongoing and vigilant postpartum monitoring up to 6 weeks postpartum is critical. Lastly, considering the hereditary nature of BSS, neonatal screening is of paramount importance to ensure early detection and timely management. As our understanding of BSS continues to expand, so will our ability to provide improved, patient‐centered care and optimize outcomes for both mother and neonate.

## Author Contributions

**Monireh Yaghoubi:** methodology, writing – original draft, writing – review and editing. **Nona Sabeti:** methodology, writing – original draft, writing – review and editing. **Ezat Hajmollarezaei:** methodology, supervision.

## Funding

This work did not receive any specific grant from funding agencies in the public, commercial, or not‐for‐profit sectors.

## Ethics Statement

This study was approved by the Research Ethics Committee of Mashhad University of Medical Sciences with the ethics code IR.MUMS.REC.1403.090.

## Consent

Written informed consent was obtained from the patient for publication of this case report. A copy of the written consent is available for review by the Editor‐in‐Chief of this journal on request.

## Conflicts of Interest

The authors declare no conflicts of interest.

## Data Availability

Data sharing not applicable to this article as no datasets were generated or analyzed during the current study.

## References

[1] D. Bhadra and S. Chakraborty , “Bernard–Soulier Syndrome (BSS) With Uncontrollable Menorrhagia,” Asian Journal of Transfusion Science 14, no. 1 (2020): 93–95. 33162718 10.4103/ajts.AJTS_61_18 PMC7607975

[2] Z. Kaya , “Bernard–Soulier Syndrome: A Review of Epidemiology, Molecular Pathology, Clinical Features, Laboratory Diagnosis, and Therapeutic Management,” Seminars in Thrombosis and Hemostasis 51, no. 2 (2024): 209–218. 39191409 10.1055/s-0044-1789184

[3] R. K. Andrews and M. C. Berndt , “Bernard–Soulier Syndrome: An Update,” Seminars in Thrombosis and Hemostasis 39, no. 6 (2013): 656–662. 23929303 10.1055/s-0033-1353390

[4] M. Hasanzad , “Bernard–Soulier Syndrome (BSS) & Tuberculosis: A Case Report,” International Journal of Mycobacteriology 3, no. 4 (2014): 283–285. 26786628 10.1016/j.ijmyco.2014.10.006

[5] M. H. Almomani and A. Mangla , Bernard–Soulier Syndrome (StatPearls Publishing LLC, 2025). 32491603

[6] E. Sarıdoğan , T. Onat , S. Arda Düz , et al., “Bernard–Soulier Syndrome From the Perspective of the Obstetrician: A Case Report With a Review of the Literature,” Zeitschrift für Geburtshilfe und Neonatologie 227, no. 3 (2023): 168–178. 36889343 10.1055/a-2024-0819

[7] G. Girardi and A. A. Bremer , “The Intersection of Maternal Metabolic Syndrome, Adverse Pregnancy Outcomes, and Future Metabolic Health for the Mother and Offspring,” Metabolic Syndrome and Related Disorders 20, no. 5 (2022): 251–254. 35384734 10.1089/met.2021.0124 PMC9360170

[8] R. A. F. T. Agha , C. Sohrabi , G. Mathew , and for the SCARE Group , “The SCARE 2023 Guidelines for Surgical Case Reports: SCARE Group,” (2023), https://www.scareguidelines.com/wp‐content/uploads/2023/02/SCARE‐2023‐Checklist.docx.

[9] M. B. Macêdo , J. M. M. Brito , P. S. Macêdo , and J. A. Brito , “Primigravida With Bernard–Soulier Syndrome: A Case Report,” BMC Research Notes 8, no. 1 (2015): 178. 25928053 10.1186/s13104-015-1145-5 PMC4434533

[10] U. Demirci , E. A. Erbilen , E. G. Ümit , C. İnan , N. C. Sayın , and A. M. Demir , “Successful Pregnancy and Delivery Management in a Patient With Bernard–Soulier Syndrome,” Obstetric Medicine 16, no. 3 (2023): 203–205. 37719997 10.1177/1753495X211067119 PMC10504883

[11] E. J. Huisman , N. Holle , M. Schipperus , et al., “Should HLA and HPA‐Matched Platelet Transfusions for Patients With Glanzmann Thrombasthenia or Bernard–Soulier Syndrome Be Standardized Care? A Dutch Survey and Recommendations,” Transfusion 64, no. 5 (2024): 824–838. 38642032 10.1111/trf.17824

[12] K. Kaushansky , J. T. Prchal , L. J. Burns , M. A. Lichtman , M. Levi , and D. C. Linch , Williams Hematology, 10e (McGraw‐Hill Education, 2021).

